# The therapeutic potential of neurofibromin signaling pathways and binding partners

**DOI:** 10.1038/s42003-023-04815-0

**Published:** 2023-04-20

**Authors:** Juan Báez-Flores, Mario Rodríguez-Martín, Jesus Lacal

**Affiliations:** 1 Laboratory of Functional Genetics of Rare Diseases, Department of Microbiology and Genetics, University of Salamanca (USAL), 37007 Salamanca, Spain; 2 Institute of Biomedical Research of Salamanca (IBSAL), 37007 Salamanca, Spain

**Keywords:** Growth factor signalling, Cell division, Cellular neuroscience, Diagnostic markers

## Abstract

Neurofibromin controls many cell processes, such as growth, learning, and memory. If neurofibromin is not working properly, it can lead to health problems, including issues with the nervous, skeletal, and cardiovascular systems and cancer. This review examines neurofibromin’s binding partners, signaling pathways and potential therapeutic targets. In addition, it summarizes the different post-translational modifications that can affect neurofibromin’s interactions with other molecules. It is essential to investigate the molecular mechanisms that underlie neurofibromin variants in order to provide with functional connections between neurofibromin and its associated proteins for possible therapeutic targets based on its biological function.

## Abstract (web summary)

Neurofibromin’s signaling pathways, post-translational modifications and key residues are explored to better understand its function and potential therapeutic targets.

## Introduction

Neurofibromatosis type 1 (NF1) (OMIM#162200) is an autosomal dominant multisystemic disorder with a worldwide incidence of approximately 1 in 3000 individuals caused by germline mutations in the *NF1* tumor suppressor gene^1^. The *NF1* gene encodes neurofibromin, a multifunctional protein capable of regulating multiple signaling pathways including Ras/MAPK^2^, Raf/MEK/ERK^3^, PI3K/AKT/mTOR^4^, Rho/ROCK/LIMK2/cofilin^5^, PKA-Ena/VASP^6^ and cAMP/PKA^7^. As a consequence, neurofibromin regulates a wide variety of cellular processes such as proliferation, migration, differentiation, cytoskeletal dynamics, apoptosis and stress responses^8^. As neurofibromin regulates the Ras/MAPK pathway, NF1 is included among the RASopathies, a group of developmental disorders caused by germline mutations in genes encoding components of the Ras/MAPK pathway^9^.

Neurofibromin can be found in human cells in two major isoforms: Isoform I (2818 residues) and Isoform II (2839 residues) and is ubiquitously expressed in most tissues; however, it is highest in the central nervous system, especially in neurons, oligodendrocytes, peripheral nerve trunks, glial cells, astrocytes, leukocytes, adrenal medulla and Schwann cells^10,11^. Despite the fact that the NF1 phenotype is variable, due to epigenetics, stochastic events and genetic modifiers^12^, it is characterized by café-au-lait macules (CALMS)^13^, skinfold freckling^14^, Lisch nodules^15^, optic pathway gliomas^16^ and neurofibromas^17^. Other symptoms include but are not limited to skeletal abnormalities, vascular injuries, learning disabilities, attention deficit, increased susceptibility to autism and social and behavioral problems^18^ and conferral of drug resistance in cancer therapy^19^. Hematopoietic neoplasms, such as juvenile myelomonocytic leukemia^20^ and the presence of pheochromocytoma^21^, are also associated with clinical manifestations in NF1 patients. The NF1 clinical observations suggest that the *NF1* gene is a critical regulator of brain neuronal function^22^, embryonic development^23^, pneumothorax and cardiovascular defects^23^, as well as a common driver gene in several aggressive human sporadic malignancies not associated with NF1^24^, including glioblastoma^25^, melanoma^26^, ovarian carcinoma^27^, lung cancer^28^, cholangiocarcinoma^29^, breast cancer^30^, lymphoblastic leukemia^31^ and other types of tumors^32^. To date, according to COSMIC database, more than 5524 different somatic variants in the *NF1* gene have been identified in human tumors, whether they are pathogenic or benign is not known. The correlation between specific genetic variants and the manifestation of different tumors in NF1 has been extensively studied; The presence of *NF1* microdeletions has been found to be associated with severe forms of NF1 and a higher risk of developing MPNST^33^, deletion of Met 992 (c.2970-2972 delAAT) has been identified as a causative factor for benign forms of NF1^34^, and the R1809C mutation has also been shown to be associated with benign symptoms^35^. Missense mutations in the region between amino acids 844–848 of the cysteine/serine-rich domain (CSRD) have been linked to more severe phenotypes, including spinal plexiform neurofibromas, optic pathway gliomas and malignant tumors^36^, and missense mutations affecting R1276 and K1423 have been linked to severe phenotypes such as cardiovascular abnormalities and spinal plexiform neurofibromas, while those affecting Met 1149 are associated with more benign symptoms^37^. There are also other studies that provide a comprehensive overview of the direct correlation between specific variants in the *NF1* gene and different tumors, and may be useful for further reading on the topic^24,38–40^. Also, an analysis was conducted on neurofibromin to identify regions that may be considered hotspots in neurofibromatosis type 1; the study found three regions within neurofibromin that were statistically significant including the RAS-GTPase domain, the CSRD, and the Armadillo1^41^. In this context, elucidation of the canonical and non-canonical effector pathways downstream of Ras activation and their ultimate cell-specific consequences has identified promising therapeutic targets^42^. These findings demonstrate the crucial role that specific genetic variants play in the manifestation of tumors in NF1, and the importance of understanding the genetic basis of NF1 for the development of new therapeutic strategies.

For several years, only the Ras-GTPase-activating protein-related domain (GRD)^43^ and the Sec14-PH domain^44^ of neurofibromin were structurally characterized. Recently, the biggest breakthrough in the field consisted in the resolution of the structure of the neurofibromin dimer. Full-length neurofibromin 3D structures were solved by a series of biochemical and biophysical experiments including size-exclusion chromatography multi-angle light scattering (SEC-MALS), small-angle X-ray (SAXS), small-angle neutron scattering (SANS) and analytical ultracentrifugation^45^. These experiments showed that neurofibromin exists as a high-affinity dimer and identified the regions important for dimerization, suggesting that neurofibromin is highly sensitive to mutations that disturb its structure^45^. After this study, several detailed molecular structures of neurofibromin by cryo-electron microscopy (cryo-EM) were reported, suggesting that the dimeric architecture rest in an equilibrium between the closed and open conformation states^46,47^. Cryo-EM reveals domain organization and structural details of the isoform 2 in either a closed, self-inhibited, zinc-ion-binding site stabilized state, or an open state^46^. In the closed conformation, HEAT/ARM core domains shield the GRD so that Ras binding is sterically inhibited. In the open conformation, a large-scale movement of the GRD occurs, which is necessary to access Ras, whereas Sec14-PH reorients to allow interaction with the cellular membrane^46^. The transition between closed and open states provides guidance for targeted studies that decipher the complex molecular mechanism behind the widespread neurofibromatosis syndrome and neurofibromin dysfunction in carcinogenesis^46^. Furthermore, the homodimer is characterized by a central lemniscate-shaped core formed by the assembly of the N- and C-HEAT domains^47^. Three-dimensional variability analysis was captured by the GRD and Sec14-PH domains positioned against the core scaffold in a closed conformation, suggesting that interaction with the plasma membrane may release the closed conformation to promote Ras inactivation^47^. Mutation or deletion at a disproportionate number of sites is likely to result in improper assembly of the dimer, contributing to the acute sensitivity of *NF1* gene to mutations in disease^47^. To date, the latest structural study that has been published using cryo-EM reveals an extended neurofibromin homodimer that has two conformational states: an auto-inhibited state with occluded Ras-binding site and an asymmetric open state with an exposed Ras-binding site^48^. This new model suggests that the GRD may interact simultaneously with two sets of Ras homodimers, but likely not a single homodimer^48^. While the occluded conformation is incompetent for Ras binding, both states of neurofibromin are compatible with the interaction of the SPRED1-EVH1 domain with the GAPex domain^48^. Also, the interaction of the Sec14-PH domain with membranes and lipids may have impacted the transition between occluded and open conformation, although the zinc-ion-binding site may be stripped out before the GRD-Sec14-PH linker is rearranged into an active conformation. It was found that nucleotide binding stimulates the active conformation of neurofibromin dimer and releases a lock that maintains an occluded inactive state, leading to the activation of the protein^48^. In addition, it was reported that a Zn2+ binding site stabilizes the dimer in a closed conformation, which is relevant to the overall conformational changes that neurofibromin undergoes upon activation^46^. These recent structures revealed a complex set of helical repeats throughout the protein, that if disrupted, are likely to affect the overall structure of the protein in a way that it interferes with the positioning of the GAP domain, probably leading to alteration of GAP activity. Despite these great advances, structural and functional insights into neurofibromin activation still remain incompletely defined. In this review, we provide functional connections between neurofibromin and its binding partners, signaling pathways and possible therapeutic targets.

### Neurofibromin domains and known interacting partners

**Fig. 1:**
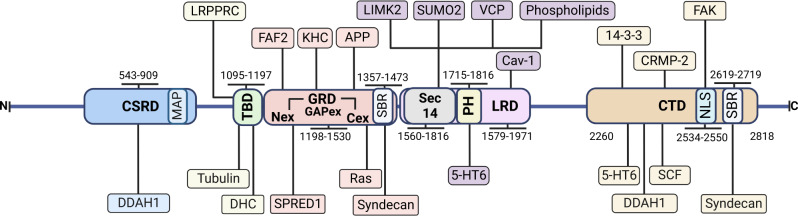
Neurofibromin domains and interacting partners. CSRD (cysteine-serine rich domain, residues 543–909), TBD (tubulin-binding domain, residues 1095–1197), GRD (GAP-related domain, residues 1198–1530), Sec14-PH (residues 1560–1816), LRD (leucine-rich domain, residues 1579–1971), the CTD (C-terminal domain, residues 2260–2818) including the NLS (bipartite nuclear localization signal domain, residues 2534–2550) and SBR (syndecan-binding region, residues 1357–1473 and 2619–2719). Domains are in bold whereas interacting partners are shown in colored boxes according to their cognate domains.

Neurofibromin contains several functional domains and regions allowing the interaction with many binding proteins and effectors (Fig. 1). The N-terminal region of neurofibromin contains several variants (R103K, D105N, M108I, L114M, E116*, A131S, and E225Rfs*6) that have been suggested to result in a non-functional protein leading to NF1 and tumor formation^49^ (Supplementary Table 1).

The cysteine/serine-rich domain (CSRD, residues 543–909) is an important allosteric activator of the adjacent GRD after PKC$\alpha$-dependent phosphorylation in neural cells^50^. In addition, it has been shown to increase the association of neurofibromin with actin upon phosphorylation, resulting in allosteric regulation of the GRD by increasing its Ras-GAP activity to arrest cell growth^50^. This region contains three cysteine pairs (622/632, 673/680, 714/721) presumably implicated in stabilizing the tridimensional structure of neurofibromin, and the highly probable palmitoylation site C845^51^ (Supplementary Table 2). Recent genetic analyses have pinpointed variants in CSRD as associated with a higher risk of developing optic pathway glioma^36,52^, plexiform and/or spinal neurofibromas, malignant neoplasm and osseous lesions^36,51^, and patients with pheochromocytoma^53^. Neurofibromin may interact with dimethylarginine dimethylaminohydrolase-1 (DDAH1), coinciding with the regions containing specific PKA phosphorylation sites^54^, whereas DDAH1 interacts with Ras^55^. DDAH1 is a nitric oxide regulator that degrades the endogenous nitric oxide inhibitor asymmetric dimethylarginine (ADMA) responsible for regulating cell proliferation^56^. Additionally, a microtubule-associated protein (MAP) domain resides within the CSRD and is thought to regulate the association of neurofibromin with microtubules^57^ (Fig. 1).

The tubulin-binding domain (TBD, residues 1095 to 1197) contains a series of 12 predicted HEAT-like repeats commonly involved in protein–protein interactions^45^ although it lacks the conserved four tandem C-terminal microtubule-binding repeats (KXGS motif)^58^ and contains sequences recognized by the proteasome (1095–1097 (KYF) and 1098–1100 (TLF))^59^. This domain interacts with $\beta$-tubulin^60^, cytoplasmic dynein heavy chain (DHC), and the leucine-rich pentatricopeptide repeat motif-containing (LRPPRC) protein^61^. The interaction with LRPPRC is of pathological interest in that it links neurofibromin to Leigh’s syndrome French Canadian (LSFC), an autosomal recessive neurodegenerative disorder that arises from variants in the *LRPPRC* gene^62^. Whereas some authors showed that TBD mediates neurofibromin dimerization^45^, this possibility has recently been ruled out^46–48^.

The GAP-related domain (GRD, residues 1198–1530) is responsible for the interaction with Ras, and it is the most well-studied functional domain of neurofibromin (Fig. 1). Neurofibromin-GRD, located central to the protein, consists of GAPex, including Nex (residues 1176 to 1247) and Cex (residues 1478–1573), and the Ras-GTPase-activating (Ras-GAP) region (1248 to 1477 residues)^63^. The Ras-GAP mediates the downregulation of the activity of all classical Ras proteins^64^, Ras-like RRAS and MRAS^65^. Indeed, when GRD is overexpressed it is sufficient to normalize the increased Ras activity and cell hyperproliferation in *Nf1*-deficient mouse cells and tissues^66,67^. It also partially restores normal phosphorylated cofilin levels and suppresses the accumulation of actin stress fibers^68^. The GRD’s Nex and Cex interact with the EVH1 domain of Spred1^63,69^, and do not impact catalysis^63^. Mutations which reside on the protein surface in the vicinity of the GRD, are more likely to directly impair or abolish the binding of this domain with Ras, whereas mutations embedded deep within the protein structure (p.R1204G and p.R1204W) may impact upon protein structure stability and abolish Ras–GRD binding indirectly^70^. Other variants (R1276E, K1423E) abolished GAP activity without reducing protein stability^48^, whereas others are dispensable for GAP activity (Supplementary Table 1). A recent precise analysis provides insight into how the membrane targeting of neurofibromin by Spred1 allows simultaneous interaction with activated KRas^71^. Other GRD interacting proteins are FAF2, APP and kinesin-1. FAF2 interacts with neurofibromin fragments (372–1552, specially from 1176 to 1552)^72^, promoting its ubiquitin-dependent proteolysis^72^. APP interacts with neurofibromin residues 1357–1557 in human melanocytes, and both proteins colocalize with melanosomes^73^ and share their interaction with the molecular motor protein kinesin-1 (Fig. 1). Kinesin-1 is required for normal distribution of mitochondria and lysosomes, and also transports cargo such as ATP along microtubules, consisting of two 120-kDa heavy chains (KHC) and two 64-kDa light chains (KLC)^74^. Neurofibromin interacts with KHC whereas APP interacts with KLC^75^. There is a syndecan-binding region (SBR, residues 1357–1473), as well as one in the CTD (residues 2619–2719) (Fig. 1). The SBR was found to mediate the interaction of neurofibromin with the cytoplasmic tail of all four mammalian syndecans^76^, transmembrane proteins that regulate signaling pathways involved in cell adhesion and migration, cellular behavior, intracellular calcium regulation and homeostasis^77^. Finally, other potential interesting regions are the Poly-Ser (1352–1355), the putative palmitoylation site C1365 and a cholesterol motif (1364–1375)^51^.

The leucine-rich domain (LRD, residues 1579–1971) consists of a glycerophospholipid binding Sec14-like domain (1560–1698), a PH-like domain (1715–1816) and part of the HEAT-like repeats (HLR) (1825–2428)^78^ (Fig. 1). It is involved in learning disabilities, skeletal problems like tibia bone defects and scoliosis^79^, as well as in inhibiting tumor metastasis and invasion of human glioblastoma cells^78^. The LRD failed to hydrolyze Ras, suggesting that its suppressive function is independent of Ras signaling^78^, and binds to caveolin-1 (Cav-1) which may act as a scaffolding protein within caveolar membranes^80^. In particular, one of the most commonly mutated positions in neurofibromin (R1809G) would impair neurofibromin function, implying a Ras-independent mechanism^48^. The SecPH binds phospholipids^44^, it also interacts with LIMK2 and would specifically prevent LIMK2 activation by ROCK^5^. However, no structural data on the complexes are available and it is not known whether this proposed mechanism actually takes place with these or other partners^81^. The serotonin 5-HT_6_ receptor, one of the several GPCRs for serotonin, activates cAMP formation on agonist stimulation and was found to interact with neurofibromin PH and CTD domains^82^. The residues K1634 and K1731 within the SecPH domain have been identified as minor and major SUMO-conjugation sites, respectively, and are hypothesized to play a critical role in the function of neurofibromin^83^. Specifically, SUMOylation of K1731 has been shown to modulate the Ras-GAP activity of the GRD domain, which is located adjacent to the SecPH domain. Additionally, it has been observed that a K1731R mutation negatively impacts the Ras-GAP activity of GRD-SecPH fragment of neurofibromin, suggesting that this site is critical for the proper functioning of neurofibromin^83^.

The HEAT-Like Repeat (HLR, residues 1825–2428) contains the structurally related Armadillo (ARM) superfamily regions 1849–1886 and 1920–1984^78^ (Fig. 1). These repeats are comprised of short hydrophobic α-helical hairpins that stack on top of each other to form long super-helical structures usually involved in protein–protein interactions^78^. Variants in this domain have been linked to NF1, suggesting an importance of the entire helical repeat scaffold for neurofibromin function^84^, including a lower risk for optic pathway glioma in NF1 patients^52,85^ (Supplementary Table 1).

The C-terminal domain (CTD, residues 2260–2818), contains a third Armadillo region (residues 2200–2571), the nuclear localization site (NLS) (residues 2534–2550)^86^ and the tyrosine kinase recognition sites (TRS) (residues 2549–2556)^87^ (Fig. 1). The CTD regulates cAMP via G-protein-dependent activation of adenylyl cyclase^88^, and has been shown to regulate the transition from metaphase to anaphase^89^, acting as a tubulin-binding domain, and to regulate nuclear localization through phosphorylation on S2808 a residue adjacent to a nuclear localization signal in the CTD by PKC-ε in glioblastoma cells, resulting in a predominantly nuclear localization^60^. Consequently, this phosphorylation could be responsible for neurofibromin translocation to the nucleus^60^, whereas phosphorylation by cAMP-dependent PKA (Supplementary Table 2) promotes association with 14-3-3 proteins, negatively regulating neurofibromin GAP activity^55^. It has been demonstrated a physical interaction between the C-terminal domain of neurofibromin and the N-terminal domain of FAK^90^, which also binds to the TPPKM motif present in the NLS (KRQEMESGITTPPKMRR) of neurofibromin^90^. The differential Microtubule-Associated-Protein (MAP) properties of NLS in both the assembly of the mitotic spindle as well as faithful genome transmission have been recently discussed^91^. Other CTD interacting proteins are CRMP-2 and CRMP-4^92^ (Fig. 1). Neurofibromin directly regulates CRMP-2 phosphorylation accessibility and activity by suppressing CRMP-2-phosphorylating kinase cascades via its Ras-GAP function, regulating neurite outgrowth and dendritic filopodia formation^93^. CRMP-2 has been implicated in multiple neurological disorders, including the development of Alzheimer’s disease. In addition, neurofibromin and CRMP-2 also work together in non-neuronal cells and contribute to cell cycle control^92^.

### Signaling pathways upstream of neurofibromin

**Fig. 2:**
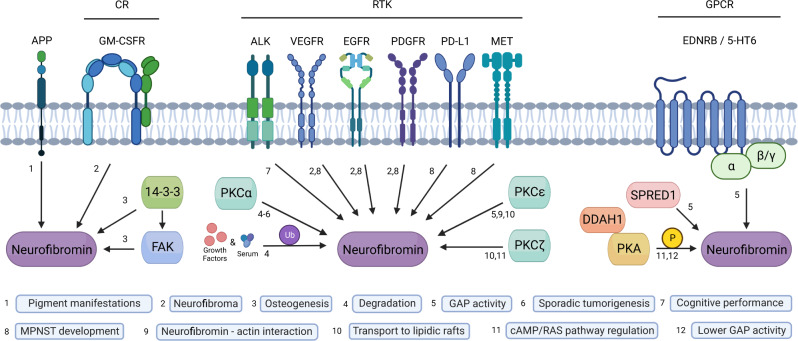
Signaling proteins upstream of neurofibromin. The APP and GM-CSFR, several RTKs and several GPCRs have been involved in neurofibromin signaling and with different biological consequences. Main cytosolic upstream regulators of neurofibromin include 14-3-3, FAK, PKC, PKA, DDAH1 and Spred1. Neurofibromin posttranslational modifications include phosphorylation and ubiquitination.

A better understanding of the implications of neurofibromin signaling functions may help to explain the diverse clinical manifestations and the increased cancer risk observed in NF1 patients. Neurofibromin is under the regulation of several upstream signaling elements including several transmembrane receptors, kinases, and cytosolic proteins (Fig. 2). Transmembrane receptors include the cytokine receptor granulocyte-macrophage colony-stimulating factor (GM-CSFR)^94^, tyrosine kinases receptors (RTKs)^95^, anaplastic lymphoma kinase (ALK)^96^, vascular endothelial growth factor (VEGFR)^97^, epidermal growth factor (EGFR)^98^, platelet-derived growth factor (PDGFR)^99^, hepatocyte growth factor (MET), GPCRs endothelin B (EDNRB)^100^ and serotonin 5-HT_6_ receptor^82^ (Fig. 2).

Receptor tyrosine kinase APP is expressed in many tissues and concentrates in the synapses in neurons where it performs physiological functions relevant to neurite growth, neuronal adhesion and axonogenesis, including kinesin-mediated axonal transport of β-secretase and presenilin-1^101^. APP and neurofibromin colocalize with melanosomes^73^, perhaps, as part of a melanosome transport/biogenesis regulating mechanism (which could be related to the etiopathogenesis of pigment-cell-related manifestations in NF1) or a mechanism for sequestering neurofibromin from the plasma membrane^73^. Another receptor upstream of neurofibromin is the GM-CSFR, which is necessary to maintain JMML in *Nf1*-mutant mice and in neurofibroma formation after nerve injury^94^. ALK was identified as an upstream activator of neurofibromin*-*regulated Ras signaling in *Drosophila* and it is responsible for several *dNf1* defects, including cognitive performance^96^. VEGFR is known to be expressed in cutaneous neurofibromas and MPNST, where it correlates with poor patient prognosis. The PDGFR is another RTK; overexpression of wild-type PDGFR associated with neurofibromin deficiency leads to aberrant activation of downstream Ras signaling and thus contributes importantly to MPNST development, indeed, it is overexpressed in Schwann cells derived from neurofibromas and MPNST^99^. Overexpression of PDGFR cooperates with loss-of neurofibromin and p53 to accelerate the molecular pathogenesis of MPNST^99^. MET has been reported activated in some MPNSTs, and implicated in resistance to Ras pathway inhibition in several cancers, including melanoma and colorectal cancer^102^. Also, the expression levels of MET and its ligand hepatocyte growth factor correlate with MPNST progression^103^.

Serum and growth factors trigger the rapid ubiquitination and complete proteasomal degradation of neurofibromin in many cell types^59^ (Fig. 2). Specifically, the literature has demonstrated that PKC plays a critical role in promoting Ras activation by destabilizing neurofibromin^104^. Additionally, research has indicated that PKCδ and α/β are essential components for maintaining the aberrant Ras signaling and promoting cell viability in neurofibromin-deficient cells^105^. Furthermore, PKCε-dependent H-Ras activation involves the recruitment of the RasGEF SOS1 and the Ras-GAP neurofibromin to the lipid rafts of embryonic neurons^51^. Lastly, neurofibromin has been shown to regulate atypical PKC activity in a RAS-dependent manner, implicating PKCz as a potentially novel effector of neurofibromin/Ras signaling in the brain^22^.

Other cytosolic upstream proteins include Spred1^69^, DAAH1^55^, the GPCR-activated protein Gβγ subunits^106^ and 5-HT_6_ receptor. Phosphorylation of S105 on Spred1 by an oncogenic RTK (EGFR^L858R^) disrupted the binding of Spred1 and neurofibromin, and as a consequence blocked negative regulation of Ras-GTP^71^. DDAH1 may interacts with CSRD and CTD and facilitates phosphorylation on these domains by PKA, T586, S818 and S876 on CSRD and some residues from 2620 to 2818 on CTD^54^. DDAH1 also exerts effects on cyclin D1 and cyclin E expression through multiple mechanisms, including VEGF, the NO/cGMP/PKG pathway, the Ras/PI3K/AKT pathway, and *NF1* expression^107^. 14-3-3 interacts with CTD upon phosphorylation by PKA, an interaction that could decrease NF1-GAP activity^55^. Finally, neurofibromin promotes 5-HT_6_ constitutive activation of Gαs/AC pathway in striatal neurons^82^. Both *NF1* silencing and NF1 patient variants within the PH domain inhibited constitutive receptor activity on the Gαs/AC pathway and reduced basal cAMP levels^82^.

### Signaling pathways downstream of neurofibromin

One of the principal functions of neurofibromin consists in the modulation of the Ras/MAPK pathway^2^, although other signaling pathways have been studied including the Raf/MEK/ERK^3^, PI3K/AKT/mTOR^4^, Rho/ROCK/LIMK2/cofilin^5^, PKA-Ena/VASP^6^ and cAMP/PKA pathways^7^.

### Ras signaling pathway

**Fig. 3:**
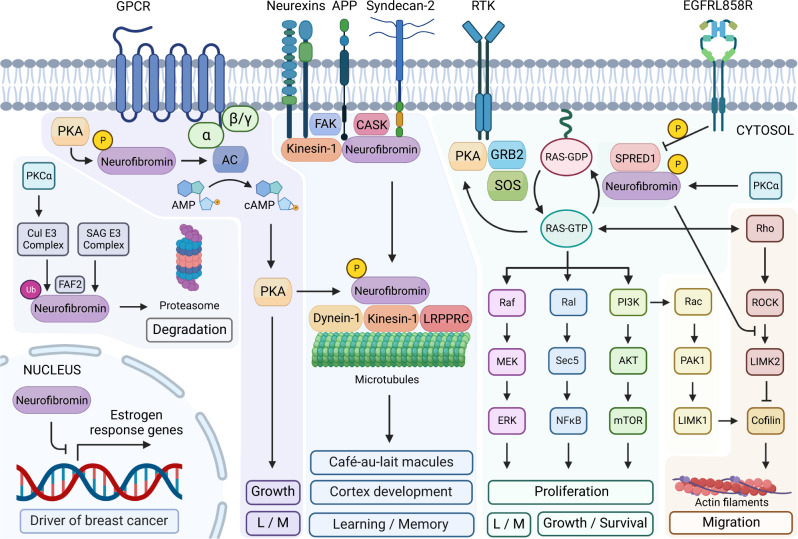
Signaling pathways downstream of neurofibromin. Neurofibromin is involved in several cell signaling pathways, including the Ras/MAPK, Akt/mTOR, Ral, ROCK/LIMK/cofilin, and cAMP/PKA pathways. From the cytosol to the nucleus neurofibromin regulates many fundamental cellular processes, such as expression of estrogen response genes, proliferation, migration, cortex development, learning and memory (L/M).

Neurofibromin enhances the rate at which the GTP-bound form of Ras is converted into the inactive GDP-bound form^2,108^. Therefore, loss-of-function mutations in neurofibromin result in the accumulation of Ras in the GTP-bound state. GTP-bound Ras proteins activate fundamental signaling pathways involved in several cellular processes such as cell polarity, proliferation, differentiation, adhesion, migration and apoptosis^109^. For instance, neurofibromin is the main Ras inactivator in dendritic spines of hippocampal pyramidal neurons^110^, long-term potentiation and hippocampal-dependent learning in interneurons^111^. Ras-GTP signals through three main molecular pathways, namely, the Raf/MEK/ERK, the Ral/NFkB and the PI3K/AKT/mTOR (Fig. 3).

The Raf/MEK/ERK pathway, as well as the PI3K/AKT/mTOR pathway, are both well-characterized pathways downstream Ras^112,113^. Both pathways are hyperactivated in neurofibromin-deficient cells, although the dependence on either MAPK or AKT signaling differs from cell type to cell type. For instance, neurofibromin-deficient hematopoietic cells are more dependent on neurofibromin/MAPK pathway growth control^114^, whereas the proliferation of neurofibromin-deficient glial cells (Schwann cells and astrocytes) primarily relies on neurofibromin/AKT signaling^115^. Hypo and hyperactivation of Ras/Rap signaling impair the capacity of synaptic plasticity, underscoring the importance of a “happy-medium” dynamic regulation of the signaling behavior^116^.

In the absence of neurofibromin, aberrant mTOR activation depends on Ras and PI3K, mediated by AKT phosphorylation and inactivation of tuberin^4^. The mechanism that involves AKT, the TSC complex and Rheb is the primary mechanism through which PI3K signaling activates mTORC1^117^. In lysosomes, neurofibromin negatively regulates mTOR in a LAMTOR1-dependent manner^118^. Ral-GEFs have also been implicated downstream of Ras as a novel cell signaling abnormality in MPNSTs^119^. Indeed, neurofibromin regulates EMT transition in a way that downregulation of neurofibromin encourages the EMT transition^120^. The EMT factors including SNAIL, SLUG, TWIST-1 and ZEB have been shown to be upregulated in MPNST deficient for neurofibromin^120,121^.

### PKA-enabled/vasodilator-stimulated phosphoprotein (PKA-Ena/VASP)

Neurofibromin modulates PKA-Ena/VASP pathway, implicated in the formation of filopodia and dendritic spine^6^, axonal outgrowth^122^, actin polymerization along bundle formation^123^, and probably for proper differentiation of nerve cells^6^. In part due to neurofibromin association with microtubular and microfilamentous cytoskeleton^91^, FAK^90^ and syndecan-2^76^, the normal development of the cerebral cortex may be affected^74^. FAK is one of the main proteins localized at focal adhesions, playing an essential role in regulating cell migration, adhesion, spreading, reorganization of the actin cytoskeleton, formation and disassembly of focal adhesions and cell protrusions, cell cycle progression, cell proliferation and apoptosis^90^. It also plays a role in osteogenesis and differentiation of osteoblasts^124^. Syndecan-2 interacts with neurofibromin and CASK, forming a multidomain scaffolding protein including also APP^73^ and neurexins^125^, while also having a role in synaptic transmembrane protein anchoring and ion channel trafficking. Indeed, it shows overlapping distribution at synaptic junctions, suggesting a potential role of neurofibromin in adhesion and signaling at neural synapses^126^. Therefore, deregulation of postsynaptic Ras signaling may explain learning disabilities associated with NF1^110^. Recent findings indicate a reciprocal regulation of Wnt/β-catenin signaling pathway and APP processing involving a physical interaction between APP and β-catenin^127^. Furthermore, APP is implicated in the pathogenesis of Alzheimer disease, as well as in cancer development in breast, pancreas, prostate and non–small cell lung cancers^128^.

### Rho/ROCK/LIMK2/cofilin pathway

Neurofibromin enhances cell motility by regulating actin filament dynamics via the inhibition of the Rho/ROCK/LIMK2/cofilin pathway^68^ (Fig. 3). In neurofibromin-depleted cells, RhoA hyperactivates ROCK and its downstream effector LIMK1/2, which continued sustained phosphorylation and inactivation of cofilin, leading to enhanced cell migration and invasion^68^. Actin depolymerizing by cofilin is a mechanism that may be one of the responsible for the neurofibroma formation in patients with NF1^68^.

### Rac1/PAK1/LIMK1/cofilin pathway

Neurofibromin also acts as a negative regulator of the Rac1/PAK1/LIMK1/cofilin pathway independently of Ras signaling pathways^68^ (Fig. 3). LIMK1 is phosphorylated and activated by PAK1, downstream of Rac1 and Cdc42 GTPases^68^. Cells with constitutively activated Rac exhibit dramatic increase in membrane ruffling, with an increase in actin polymerization and formation of stress fibers^129^. Neurofibromin involvement in these signaling pathways has been established. However, most of its molecular targets are still unknown, and the molecular mechanisms remain in most cases to be elucidated^64^.

### cAMP/PKA pathway

Neurofibromin is a positive regulator of intracellular cAMP levels (Fig. 3), which cannot be reversed by inhibition of Ras-MEK or Ras-PI3K downstream signaling^130^. The cAMP/PKA pathway is involved in many processes, such as inhibition of cell growth^131^, induction of apoptosis^132^, arrest of the rearrangement of the cytoskeleton^133^, neuropeptide responses^134^, development and functioning of the nervous system, synaptogenesis^6^, learning process and memory shaping^135^, sugar and lipid metabolism^136^ and cancer^137^. The stimulation of GPCR causes neurofibromin to regulate cAMP production in CNS neurons through different Gα activators, not Gαi. This regulation is dependent on Ras and results in interaction with target PKA^22^ (Fig. 3). Ras/cAMP regulation operates through the activation of atypical PKCz, leading to GRK2-driven Gα inactivation^22^. These observations highlight the regulation of a diverse number of GPCRs in distinct CNS cell population, suggesting a potential strategy to correct NF1-related CNS deficits^22^.

In NF1 melanocytes, the molecular mechanisms of melanin synthesis, if NF1 is inactivated, are linked to increased activity of cAMP-mediated PKA and ERK signaling pathways, which in turn leads to overexpression of the key transcription factor MITF and melanogenic enzymes, such as tyrosinase and TRP-2/dopachrome tautomerase, resulting in hyperpigmentation^138^. Moreover, the overexpression of cAMP-responsive element-binding protein (CREB) bound to the brain-enriched microRNA-9 promoter has been described to repress expression of *NF1*, and encourage cell migration^139^. Interestingly, miR-514a overexpression was correlated with increased melanoma cell resistance to BRAFi through decreased expression of *NF1* and associated with pairing therapies involving target-based therapy^140,141^.

A *Drosophila Nf1* model revealed that neurofibromin is essential for the cellular response to neuropeptides, like pituitary adenylate cyclase activating polypeptide-38 (PACAP38) at the neuromuscular junction, through activation of the cAMP/PKA pathway^134^. Human PACAP38 activates the receptor for insects PDF when co-expressed with neurofibromin, potentiating PDF action by coupling to AC^142^, and induces cell growth in astrocytes activating MAPK^143^. In neuroblastoma cells, PACAP38 regulation is mediated by PAC1 receptor through a cAMP-dependent but PKA-independent mechanism^144,145^.

### The ubiquitin–proteasome pathway

Neurofibromin is dynamically regulated by the ubiquitin–proteasome pathway, triggered by several growth factors which reduced neurofibromin levels rapidly in a variety of cell lines^59^. This regulation appears to be independent of Ras activation, as exogenous expression of an activated Ras allele did not induce neurofibromin degradation, and inhibitors of MEK and PI3K did not prevent degradation^59^. Neurofibromin is a physiological substrate of the SAG E3 ubiquitin ligase during embryogenesis^146^. Also, the Cul E3 complex and the BTB adaptor protein KBTBD7 regulate PKC-mediated neurofibromin ubiquitination in normal and pathogenic settings^147^. The hypoxia-associated factor (HAF) might also promote ubiquitination and proteasomal degradation of neurofibromin, which may be significant during physiological hypoxia including embryonic development and wound healing, but may also play a driving role in the development and progression of hypoxia-associated cancers^148^.

### Neurofibromin as an estrogen-receptor (ER) transcriptional co-repressor in breast cancer

The correlation between *NF1* loss and upregulation of ER-associated pathways in human breast cancer^149^, increased tumor aggressiveness and poor patient prognosis, associate neurofibromin with breast cancer^149^. In addition, *NF1* has been reported to be mutated more frequently in ER^+^ metastatic breast cancer, suggesting it is a driver of breast cancer progression^150^. Recently, it has been demonstrated that neurofibromin acts as a dual repressor for both Ras and ER signaling; co-targeting may treat neurofibromin-deficient ER^+^ breast tumors^151^.

### Neurofibromin-HIPPO

**Fig. 4:**
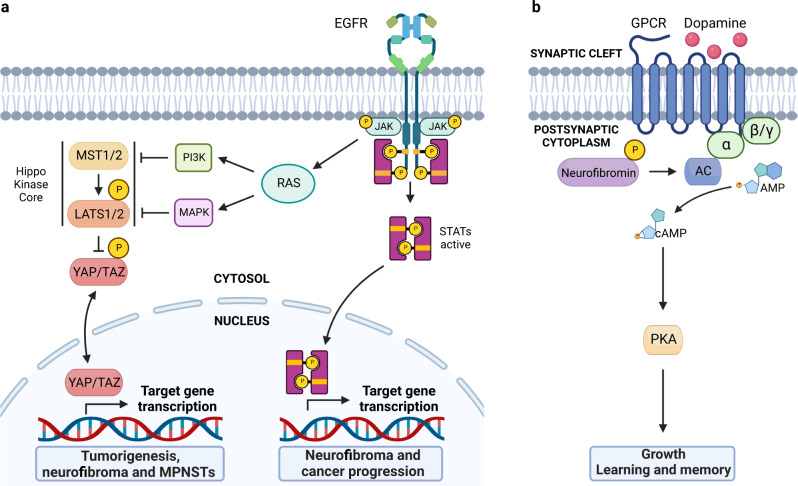
Hippo, JAK/STAT and dopamine signaling pathways are regulated by neurofibromin. **a** The Hippo pathway is a master regulator of tissue homeostasis and organ size in which MST1/2, LATS1/2 and YAP/TAZ are major players. The JAK/STAT pathway regulates embryonic development and is involved in processes such as stem cell maintenance, hematopoietic and inflammatory response. These pathways also regulate gene expression implicated in tumorigenesis and cancer progression. **b** This pathway involves neurofibromin in regulating cAMP levels, important in neuronal connections in which dopamine travels to areas of the brain and body to convey important information such as executive thinking, cognition, feelings of reward and pleasure and voluntary motor movements. Dopamine is thought to guide learning via dynamic and differential modulation of PKA.

HIPPO signaling is known to regulate a variety of cellular processes including cell cycle progression, apoptosis, tissue regeneration, cell differentiation, and the control of organ size and development. In this pathway MST1/2 kinases activate LATS1/2 kinases, which in turn phosphorylate and inhibit the nuclear translocation of transcriptional coactivators YAP and TAZ, regulating gene expression^152^ (Fig. 4a). Dysregulation of the HIPPO pathway contributes to cancer development through tumorigenesis^153^ and cutaneous neurofibromas from NF1 patients^154,155^. On the other hand, MPNSTs show an elevated HIPPO-YAP/TAZ expression^156^. Remarkably, the YAP signature is present in MPNSTs regardless of their *NF1* genetic status, suggesting that activation of the HIPPO-YAP/TAZ pathway is common to both genetic and sporadic MPNSTs^156^. YAP and TAZ directly interact with JUNB and STAT3 via a WW domain important for transformation, and they stimulate transcriptional activation by AP-1 proteins, implicated in the development and maintenance of cancers^157^.

### JAK2/STAT3 signaling pathway

This signaling pathway can influence the transcription and expression of multiple genes involved in biological processes such as cellular growth, metabolism, differentiation, degradation and angiogenesis (Fig. 4a). The JAK-signal transducer and STAT members, particularly STAT3, have been demonstrated to be very important for cancer progression^158^. EGFR is one of the receptor tyrosine kinases that mediates STAT3 phosphorylation and is expressed in most human MPNST^159^. In addition to the JAK-signal transducers and activators of transcription axis, JAKs can also affect other signaling pathways through intracellular crosstalk, highlighting the Ras pathway^160^.

### Neurofibromin and dopamine

Neurofibromin is a positive regulator of dopamine homeostasis, as *NF1* variants in human and mouse neurons lead to reduced levels of the neurotransmitter dopamine^161,162^. Using behavioral, electrophysiological and primary culture, it has been demonstrated that reduced dopamine signaling is responsible for cAMP-dependent defects, whereas pharmacological elevation of dopamine reverses in neuron function, learning, memory and attention deficits in *NF1*-mice^163^ (Fig. 4b). A dose-dependent relationship was identified between neurofibromin levels, dopamine signaling and cognitive deficits in the hippocampus and striatum on NF1 patients^164^.

### Neurofibromin therapeutic targets

**Fig. 5:**
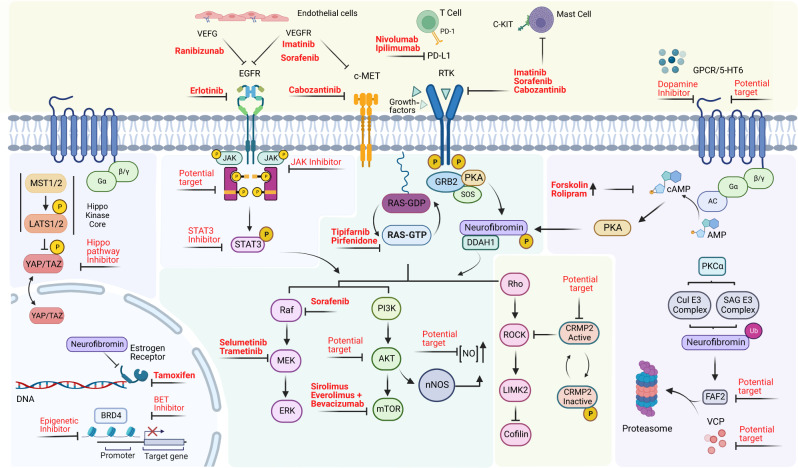
Neurofibromin therapeutic inhibitors and potential targets. The identification of key regulatory neurofibromin partners may have important clinical implications, in a strategy aimed at blocking its inactivation and/or upregulating the protein. These benefits may extend beyond therapies relevant to neurofibromin to serve as a potential clinical strategy to attenuate the Ras pathway in tumors harboring variants in genes that function upstream of Ras. Inhibitors and potential targets are shown in red.

When altered, neurofibromin has been involved in neurofibromatosis type 1, highly aggressive malignant diseases and mechanisms of treatment resistance. In that context, several studies have reviewed the potential therapeutics, the mechanism of action, as well as the information on the status of how far a drug has progressed clinically^42,165–168^. In this work, we have summarized all therapies targeting the upstream/downstream molecules of neurofibromin with potential to become novel strategies for the treatment of NF1-related malignancies (Fig. 5). Attractive therapeutic targets include: the modulation of nitric oxide^54,107^, histone deacetylase inhibitors^169^, YAP and TAZ^156^, inhibition of VEGFR2/MET/RET by cabozantinib in MPNST^102^, the HIPPO pathway^152,155^ and Ral-GEFs for MPNSTs^119,170^, CRMP-2 for therapeutic intervention in patients with NF1 pain^171^, ALK^172^, inhibition of FAF2^72^ and STAT3 on neurofibromas^173^, and VCP^174^ for NF1-cancer therapy, 5-HT_6_ receptor for cognitive impairment^175^ and neurofibromin-mediated cAMP production^176^.

MPNST therapies include nivolumab and ipilimumab (Phase II clinical trial)^177^, inhibition of BRD4 with CPI-0610 (Phase II clinical trial) triggering apoptosis through Bim^178^, and a combination of a BET, MEK, and PD-L1 receptor inhibitors^166^. Recently, a NF1 patient treated with tofacitinib, a selective JAK1/JAK3 inhibitor that regulated neurofibromas growth^179^, as well as their progression to MPNST, has been described^159^. On the other hand, the combination of everolimus and bevacizumab did not achieve favorable results in studies in patients with MPNST^180^. Likewise, neither targeting VEFG-A using ranibizumab, nor targeting VEFGR with sorafenib or imatinib, were effective for cutaneous neurofibromas or MPNST respectively^181^. EGFR is abundantly expressed in neurofibroma and MPNST cell lines, although unfortunately the EGFR inhibitor erlotinib failed to inhibit tumor growth in Phase 2 trials of patients with advanced-stage MPNST^166^. Forskolin and rolipram have also been used for impaired cAMP production, in particular, rolipram inhibited optic glioma growth and tumor size^162,182^. Everolimus is well suited for future consideration as initial therapy in patients with low-grade pediatric glioma^183^. Immune checkpoint inhibitors/therapies include targeting PD-1 and PD-L1^184^, although immunotherapy with monoclonal antibodies remain to be identified in NF1 preclinical models^185^. The proteasomal regulation of neurofibromin represents an important mechanism of controlling both the amplitude and duration of Ras-mediated signaling, which may be exploited therapeutically by promoting degradation of neurofibromin^146,186,187^.

Preclinical trials suggest that dopamine-target therapies, such as methylphenidate, a stimulant medication that increases dopaminergic and noradrenergic neurotransmission, may be useful treatments for children with NF1-associated cognitive abnormalities. However, the therapeutic effect on cognitive performance is unclear^188^. Nevertheless, targeting VEGF-A suggested a qualitative improvement in vision after bevacizumab-based treatment in children with OPGs^189^.

When expression of *NF1* is inhibited, the resulting ER^+^ breast cancer cells were stimulated by tamoxifen (a drug commonly used to prevent relapses from ER^+^ breast cancer) instead of inhibited, and these cells became sensitive to a very low concentration of estradiol^151^. Breast cancer patients with *NF1* sporadic mutations treated with the estrogen-receptor antagonist fulvestrant showed a good outcome^190^. Combination with CDK4/6 inhibitors, which target ER-independent cyclin D1 transcription, results in substantially enhanced efficacy of endocrine therapy in vitro^190^. These findings suggest that the prognosis of patients with pretreatment detection of *NF1* mutation in the PALOMA- phase III trial^191^ could overcome the risk of early relapse via the investigation in an adjuvant setting in *NF1*-mutant cancers of combined fulvestrant and palbociclib^192^.

Inhibitors for the major signaling pathways related to neurofibromin have also been studied. Ras inhibitors include tipifarnib and pirfenidone, although they did not significantly prolong the time to progression compared with placebo in children and young adults with NF1 and progressive plexiform neurofibromas^193^. Inhibitors for the Raf/MEK/ERK pathway include the MEK inhibitor trametinib^194^ and selumetinib, which resulted in shrinkage in neurofibromin-related gliomas in the optic pathway^195^, becoming the first FDA-approved treatment for inoperable plexiform neurofibroma^196^. The mTOR pathway has been investigated as a potential therapeutic target^180^, including the inhibitor sirolimus (rapamycin) which did not provide any favorable results of shrinkage of the plexiform neurofibroma, although it could delay their growth in selected patients^197^.

### Conclusions

Neurofibromin is ubiquitously expressed with enrichment in neurons, Schwann cells, oligodendrocytes, astrocytes, leukocytes and adrenal medulla, and it is highly conserved among species. Due to its high degree of conservation, several animal models can be used to identify potential effectors, partners, and promising therapeutic targets to complete the functional characterization of this protein. Neurofibromin is known to associate with a large number of proteins, including transmembrane receptors, soluble effectors, proteins at the cell surface, the cytoskeleton and the nucleus. Although the biological significance of these protein–protein interactions is largely unknown, there is profound evidence on neurofibromin roles in actin cytoskeleton remodeling, cell motility, cell adhesion, proliferation, differentiation, apoptosis, stress responses, learning and memory. Variants throughout the protein affecting different domains that interact with different binding partners may be associated with the vast array of clinical manifestations. Some neurofibromin effectors have been verified, whereas many reported interactions remain unsubstantiated and may be irrelevant. The diversity of protein associations does however emphasize the point that neurofibromin is likely to act through the canonical and non-canonical effector pathways downstream of Ras activation. Several groups have reviewed neurofibromin protein structure, putative interacting partners and therapeutic strategies^42,198–201^, but to date, a high-quality NF1 interactome has not been described yet. The fact that neurofibromin has been related to a variety of membrane receptors and that binding partners may be cell type-specific makes elucidating its additional binding partners and functions even more intriguing. Further studies on the regulation of neurofibromin in various model organisms and cell types are needed in order to identify the role of neurofibromin under pathological conditions.

## Supplementary information

Supplementary Information
