# Supplementary material for: The therapeutic potential of neurofibromin signaling pathways and binding partners
Source: Commun Biol. 2023 Apr 20;6:436. doi: 10.1038/s42003-023-04815-0 (PMC10119308; doi:10.1038/s42003-023-04815-0)
Supplement: Supplementary file 1 — Supplementary Information [file 42003_2023_4815_MOESM1_ESM.pdf]

Supplementary Table 1. Neurofibromin key residues involved in protein structure and protein-protein associations.

Domain	Residue	Binding partner	Mutation	Effect in protein	Ref
N-terminal region	103	PKC	R>K	Nd	49
	105	PKC	D>N	Nd	49
	108	PKC	M>I	Nd	49
	114	PKC	L>M	Nd	49
	116	PKC	E>*	Truncated protein → loss of functional GRD and CSRD domains	49
	131	PKC	A>S	Nd	49
	225	PKC	E>Rfs/6	Truncated protein → non functional	49
Interdomain region	304	PKC	R>*	Improper functioning of PKC phosphorylation	84
	315		Q>Q		84
	316		L>M		84
	335		N>N		84
CSRD	777	Ras	W>R	Reduction of RasGAP activity	48
	844-848	DDAH1	Missense	Severe phenotypes	36
Interdomain region	991	Nd	MΔ	Reduction of protein stability	36
	1084	Nd	P>R		36
TBD	1095-1097 (KYF)	Nd	Nd	Degradation	48
	1098-1100 (TLF)	Nd	Nd		48
	1276	Ras	R>E	Reduction of RasGAP activity	48
GRD	1423		K>E		48
PH	1719-1736	5-HT6	Δ	Defects in 5-HT6 activity	82
	1746-1750		Δ		82
	1748		R>A		82
	1750		K>A , Δ		82
	1809	Ras	R>G	Reduction of RasGAP activity	48
HLR	1825-2428	Nd	Nd	Lower risk of OPG	52
	1834	NF1	L>R	Dimerization	38
	2104		L>R		48
	1849	Nd	R>Q	Dimerization<br>Reduction of protein stability	48<br>48
Interdomain region	1869	Ras	G>S	Reduction of RasGAP activity	48
	2067		L>P	Reduction of RasGAP activity and protein stability	48
CTD	T2554	14-3-3	S/T> A	Significantly decreased binding activity with 14-3-3	55
	S2576				55
	S2578				55
	S2580				55
	S2813				55

Supplementary Table 2. Neurofibromin posttranslational modifications.

Domain	Residue	Modification	Protein	Effect in neurofibromin	Ref
N-Terminal region	Nd	Phosphorylation	PKCα	Proteasomal degradation	104
N-Terminal region	Nd	Phosphorylation	Hyperactivated PKCα	Destabilization, role in sporadic tumorigenesis	104
N-Terminal region	Nd	Phosphorylation	PKCβ	Nd	105
N-Terminal region	Nd	Phosphorylation	PKCε	Recruitment to lipid rafts Increase of RasGAP activity	50, 51
N-Terminal region	Nd	Phosphorylation	PKCζ	Nd	22
CSRD	T586	Phosphorylation	PKAα	Allosteric activation of GRD domain	54
CSRD	S818	Phosphorylation	PKAα	Allosteric activation of GRD domain	54
CSRD	S876	Phosphorylation	PKAα	Allosteric activation of GRD domain	54
CSRD	C622/C632	Palmitoylation	Nd	Presumably stabilization of tridimensional structure	51
CSRD	C673/680	Palmitoylation	Nd	Presumably stabilization of tridimensional structure	51
CSRD	C714/721	Palmitoylation	Nd	Presumably stabilization of tridimensional structure	51
CSRD	C845	Palmitoylation	Nd	Nd	51
TBD	1095-1097 KYF	Ubiquitination	Nd	Degradation of the protein	59
TBD	1098-1100 TLF	Ubiquitination	Nd	Degradation of the protein	59
GRD	1176-1552	Ubiquitination	FAF2 (ETEA)	Ub-dependent proteolysis	72
GRD	C1365	Palmitoylation	Nd	Weaker RasGAP activity in GRD nf1 type II	51
SEC14-PH	K1634	SUMOylation		Role in Ras-GAP activity	83
SEC14-PH	K1731	SUMOylation		Role in Ras-GAP activity	83
CTD	T2554	Phosphorylation	PKA	Association with 14-3-3, leading to a negative regulation of GAP activity	55
CTD	Y2556	Phosphorylation	CK2	Association with 14-3-3, leading to a negative regulation of GAP activity	55
CTD	S2576	Phosphorylation	PKA	Association with 14-3-3, leading to a negative regulation of GAP activity	55
CTD	S2578	Phosphorylation	PKA	Association with 14-3-3, leading to a negative regulation of GAP activity	55
CTD	S2580	Phosphorylation	PKA	Association with 14-3-3, leading to a negative regulation of GAP activity	55
CTD	S2813	Phosphorylation	PKA	Association with 14-3-3, leading to a negative regulation of GAP activity	55
CTD	Nd	Ubiquitination	HAF	Proteasomal degradation during hypoxia	149
Nd	Nd	Ubiquitination	Cul3 E3 ligase	Proteasomal degradation	149
